# Out‐of‐home care in childhood and biomedical risk factors in middle‐age: National birth cohort study

**DOI:** 10.1002/ajhb.23343

**Published:** 2019-11-05

**Authors:** Carlos de Mestral, Steven Bell, Mark Hamer, George David Batty

**Affiliations:** ^1^ Department of Epidemiology & Public Health University College London London UK; ^2^ Center for Primary Care Medicine & Public Health University of Lausanne Lausanne Switzerland; ^3^ Department of Public Health & Primary Care University of Cambridge Cambridge UK; ^4^ Division of Surgery & Interventional Science University College London London UK; ^5^ School of Biological & Population Health Sciences Oregon State University Corvallis Oregon

## Abstract

**Objective:**

With there being an apparent impact of experience of out‐of‐home care in childhood on chronic disease and mortality, we examined how such adversity might be embodied such that it has a measurable impact on human biology, so mediating this relationship.

**Methods:**

We used data from the UK National Child Development Study in which exposure to public care was prospectively gathered on three occasions up to age 16. Study members also participated in a social survey at age 42 and a clinical examination at age 44/45 when cardiovascular, inflammatory, neuroendocrine, and respiratory risk markers for mortality were collected, 19 of which were included as endpoints in the present analyses.

**Results:**

Of the 8012 participants in the biomedical survey, 4% (*n* = 322) had been in care at some point in childhood and/or adolescence. We found the expected marked differences in the early life characteristics of poverty, health, and disability in children with experience of public care relative to their unexposed counterparts. After controlling for these confounding factors, however, care in childhood was essentially unrelated to biomarkers in middle‐age. We also found no consistent links between these biomarkers and the duration, timing, or type of care.

**Conclusions:**

Our results suggest that the biomarkers captured in the present study are unlikely to mediate the link between public care in childhood and later chronic disease or mortality. Processes involving mental health, socioeconomic position, and health behaviors would seem to be a potential alternative pathway warranting investigation.

## 1 INTRODUCTION

In both the United States and United Kingdom, there has been a secular rise in the prevalence of children placed in out‐of‐home care—also referred to as public care or looked‐after children—most typically in foster homes (U. S. Department of Health and Human Services, 2018; UK Department of Education, 2018). The large majority of these children will have been removed from their biological families in order to safeguard their welfare following parental abuse and/or neglect. Although placement in public care is predicated upon providing a more nurturing and protective environment for children than that available via their family of origin, there is some evidence that childhood out‐of‐home care is linked to poor outcomes in later life. Thus, compared with their peers, looked after children, are, as adults, more likely to engage in destructive behaviors such as substance abuse (Gypen, Vanderfaeillie, De Maeyer, Belenger, & Van Holen, 2017; Zlotnick, Tam, & Soman, 2012) and experience a marked elevation in rates of chronic illness and mortality (Gao, Brannstrom, & Almquist, 2017).

It is unclear how exposure to public care is embodied, such that it has a measureable impact on biological processes, therefore, potentially mediating the relationship with mortality risk. While there is growing evidence that childhood disadvantage, broadly defined, might have an influence on markers of low‐grade inflammation (Danese, Pariante, Caspi, Taylor, & Poulton, 2007; Packard et al., 2011), much attention has been given to cortisol levels—with discordant results (Deighton, Neville, Pusch, & Dobson, 2018)—when there are in fact no consistent links between this neuroendocrine marker and mortality or chronic disease (Baylis et al., 2013; Davey Smith et al., 2005; Kumari, Shipley, Stafford, & Kivimaki, 2011). We are unaware of any studies examining whether childhood placement in out‐of‐home care is linked with biomarkers representing the various cardiovascular and, respiratory pathways to mortality. Alongside neuroendocrine and inflammatory indices, we therefore examined these new potential relationships in adult men and women drawn from a relatively large and well‐characterized birth cohort study who took part in a medical examination in middle‐age and had prospectively collected data on early life care that, in addition to allowing us to explore the effect of being looked after, also facilitate an examination of the influence, if any, of its timing and duration. It is plausible, for instance, that being taken into care later in adolescence, as opposed to infancy when perception of environment is less acute, may have a longer term negative impact on biological risk markers. Children with a longer duration of care experience may also fair less well as adults than those who had a shorter exposure.

## 2 METHODS

Described in detail elsewhere (Batty, Ploubidis, Goodman, & Bann, 2018), we used data from the National Child Development Study (also known as the 1958 Birth Cohort Study), an ongoing UK study that sampled all births during a single week in March 1958 (*n* = 18 558). To date, participating study members have responded to surveys on 10 occasions in addition to a biomedical examination at age 44/5. Data collection according to wave and attrition is depicted in Figure S1. This study was approved by the National Health Service Research Ethics Committee.

### 2.1 Prospective assessment of out‐of‐home care

We determined participants' out‐of‐home care history in childhood from responses provided prospectively in the follow‐up surveys at ages 7, 11, and 16 years. During these surveys, parents were asked: “Has the child been under the care of the local authority, now or in the past?”. Where applicable, enquiries were also made about the child's age when first placed in care. Using these data, we created a binary variable for exposure to care (yes/no), and, in order to explore timing of exposure, the age such care began: never; 0 to 7 years; 8 to 11 years; 12 to 16 years. Other enquiries also had some utility in the present context. At the age 11, parents with a child in care were asked about the type (foster care or other); “other” is likely to constitute group or institution‐based care such as a children's home. Also, at age 23, study members who recalled having been in childhood care estimated the duration (≤1 year; 1‐3 years; and ≥3 years). Agreement between retrospective recall of ever having been in public care at age 23 and prospectively gathered data up to up age 16 years was very high (kappa statistic 0.97, *P*‐value < .001).

### 2.2 Measurement of biomedical data in adulthood

In the biomedical survey at age 44/45 years, using standard protocols, study members provided non‐fasting venous blood and saliva samples for the following risk indicators (Batty et al., 2018; Calvin, Batty, Lowe, & Deary, 2011): salivary cortisol sampled 45 minutes and again 3 hours after waking, C‐reactive protein, D‐dimer, fibrinogen, glycated hemoglobin, high‐density lipoprotein, low‐density lipoprotein, total cholesterol, triglycerides, tissue plasminogen activator, von Willebrand factor, and immunoglobulin E. We averaged three blood pressure and heart rate readings, and calculated waist‐to‐hip ratio [waist (cm)/hip circumference (cm)] and body mass index [weight (kg)/height (m)^2^] (Chandola, Deary, Blane, & Batty, 2006). Forced expiratory volume in 1 second and forced vital capacity were also measured on three occasions with the highest value used herein.

### 2.3 Collateral data

Parental socioeconomic position was based on the father's occupational social class at birth, or if missing, the mother's, as measured by the UK Registrar General's Social Class scheme (Stringhini et al., 2018). Maternal age, marital status, and educational level (continued beyond mandatory education or not) at participant's birth were also self‐reported. Data on childhood factors were collected, including hospitalizations, disability, and psychological distress as ascertained by measuring several mood and behavioral symptoms using the validated, teacher‐rated Bristol Social Adjustment Guide at ages 7 and 11 (Clark, Rodgers, Caldwell, Power, & Stansfeld, 2007). We created a continuous summary measure each for internalizing and externalizing symptoms, taking the mean of the standardized scores across the three childhood follow‐up surveys (higher scores denote more disadvantage) (Clark et al., 2007). Scores were then dichotomized using the highest quartile as the threshold. Adult health behaviors and socioeconomic position were self‐reported during face‐to‐face interviews at the age 42 survey and included alcohol consumption (heavy intake defined as ≥168 g/week for men; ≥112 g/week for women), cigarette smoking (smoker vs non‐smoker), and occupational social class (schema as above) (Gale, Johnson, Deary, Schoon, & Batty, 2009).

### 2.4 Statistical analyses

We first ascertained if there were any key differences in potential confounding and mediating variables according to childhood care status. To do so, we computed either t‐tests or chi‐squared tests. Those characteristics linked to out of home care were then included in linear regression models to compute beta coefficients with accompanying 95% confidence intervals to summarize the association of public care with each adult biomedical outcome. Selected biomedical markers (C‐reactive protein, immunoglobulin E, and both cortisol measures) had skewed distributions, common to such laboratory assays, and were therefore log transformed. With this being a cohort of births within the same week, no adjustment for age was required. In our most basic analyses we adjusted the beta coefficients for sex; this was followed by potential confounding factors which captured early life socioeconomic position, disability and health, and, lastly, the potential mediating variables of adult socioeconomic position and health behaviors. Throughout the regression analyses, the analytical sample is based on non‐missing data and is the same for different statistical models but varies across biomedical outcomes. See footnote to Table 2 for the composition of the statistical models. All analyses were conducted using Stata statistical software version 15 (StataCorp, College Station, Texas).

## 3 RESULTS

Table 1 shows the characteristics of cohort members in the mid‐life medical examination according to out‐of‐home care placement in childhood. Of the 8012 participants included in the analysis, 4% (*n* = 322) had experienced out‐of‐home care by the age of 16 years. By most early life characteristics, children who had experience of being looked after were disadvantaged. Particularly stark were the differentials in disability, mental health, and socioeconomic position. Cohort members exposed pre‐adulthood to public care were also markedly more likely to be socioeconomically deprived and to smoke cigarettes by middle‐age ‐ again, differentials were pronounced ‐ though heavy alcohol intake had a similar prevalence in both groups.

**Table 1:** Early life and adult characteristics of cohort members according to placement in out‐of‐home care in childhood, National Child Development Study (*n* = 8012)

	Out‐of‐home care in childhood		*P*‐value for difference
	Yes	Never	
Number of study members	4.0 (322)	96.0 (7690)	
Women	51.2 (165)	50.3 (3871)	.75
Early life factors			
Parental manual social class	84.8 (273)	68.9 (5299)	<.001
Maternal age at birth, mean (SD)	26.3 (5.9)	27.6 (5.6)	<.001
Mothers married	79.0 (245)	97.7 (7199)	<.001
Low maternal education	82.8 (255)	72.3 (5314)	<.001
Childhood hospitalizations	14.4 (39)	11.4 (791)	.14
Childhood disability	23.0 (73)	13.9 (1062)	<.001
High internalizing symptoms	33.9 (108)	19.5 (1498)	<.001
High externalizing symptoms	41.4 (132)	19.6 (1502)	<.001
Adult factors			
Manual social class	55.3 (178)	38.1 (2931)	<.001
Cigarette smoking	42.0 (131)	23.3 (1734)	<.001
Heavy alcohol consumption	25.7 (58)	25.9 (1606)	.93

*Note*: Results are percentage (number of participants) unless otherwise reported.

In Table 2, we summarize the association between care in childhood and adult biomedical risk factors; no history of care was the referent group. After adjustment for sex, only four of the 19 biomedical outcomes showed associations at conventional levels of statistical significance—fibrinogen, cortisol, forced expiratory volume in 1 second, and forced vital capacity—and these were of low magnitude. Of these, there was in fact a more favorable (lower) level of cortisol in adults with a history of childhood care. Being looked after was associated with both lower forced expiratory volume in 1 second and forced vital capacity with very similar magnitude, this dual relationship being unsurprising given that these endpoints are themselves strongly correlated (*r* = 0.80, *P*‐value <.001, *n* = 7697). After taking into account the early life confounding factors of poverty and health, the care‐biomedical marker associations diminished in magnitude somewhat. A weak relationship also emerged for body mass index, such that children with experience of public care appeared to be leaner in adulthood. Results of analyses where the present multivariable model was disaggregated and mediating variables then introduced, largely to explore the result for body mass index, are given in Table S1. However, our overall conclusion of essentially no relationship between childhood care and adult biomedical indices was unchanged.

**Table 2:** Association of out‐of‐home care in childhood with adult biomedical risk factors, National Child Development Study

		Mean (SD)	Analytical sample	Sex‐adjusted, β (95% CI)	*P*‐value	Confounder‐adjusted, β (95% CI)	*P*‐value
Cardiovascular disease risk factors	Resting SBP, mmHg	126.4 (16.3)	5672	−1.51 (−3.68, 0.65)	.17	−1.33 (−3.54, 0.88)	.24
	Resting DBP, mmHg	78.7 (10.7)	5671	−0.48 (−1.96, 1.01)	.53	−0.45 (−1.97, 1.07)	.56
	Resting heart rate, bpm	71.1 (11.4)	5702	0.50 (−1.12, 2.12)	.55	0.11 (−1.55, 1.78)	.90
	BMI, kg/m^2^	27.2 (4.5)	5616	−0.44 (−1.08, 0.20)	.18	−0.83 (−1.49, −0.18)	.02
	WHR	0.9 (0.1)	5716	0.00 (−0.01, 0.01)	.93	−0.01 (−0.01, 0.00)	.26
	Triglycerides, mg/L	1.9 (1.1)	4755	−0.01 (−0.18, 0.16)	.92	−0.05 (−0.22, 0.13)	.59
	HDL, mmol/L	1.5 (0.4)	4782	−0.01 (−0.07, 0.04)	.62	0.01 (−0.05, 0.06)	.75
	LDL, mmol/L	3.4 (0.9)	4543	−0.06 (−0.20, 0.08)	.39	−0.07 (−0.21, 0.08)	.35
	HbA1c, %	5.2 (0.4)	4836	0.02 (−0.03, 0.08)	.42	0.00 (−0.06, 0.06)	.92
Inflammatory markers	CRP, mg/L	1.8 (2.3)	4720	0.02 (−0.16, 0.20)	.80	−0.08 (−0.26, 0.11)	.42
	Fibrinogen, g/L	2.9 (0.6)	4723	0.12 (0.03, 0.21)	.01	0.08 (−0.01, 0.17)	.08
	D‐dimer, ng/mL	177.8 (97.6)	4665	4.42 (−10.47, 19.31)	.56	1.92 (−13.38, 17.21)	.81
	tPA, ng/mL	5.1 (2.5)	4730	0.29 (−0.10, 0.68)	.14	0.17 (−0.23, 0.57)	.40
	vWF, IU/dL	120.6 (38)	4732	0.47 (−5.53, 6.46)	.88	−1.34 (−7.51, 4.83)	.67
	IgE, mg/dL	67 (109)	4673	0.05 (−0.16, 0.26)	.64	0.00 (−0.22, 0.22)	.99
Neuroendocrine markers	Cortisol 1, nmol/L	21.3 (12.1)	4029	−0.12 (−0.22, −0.01)	.03	−0.10 (−0.21, 0.00)	.05
	Cortisol 2, nmol/L	8.5 (6.9)	4055	0.03 (−0.08, 0.14)	.61	0.00 (−0.11, 0.12)	.95
Pulmonary function	FEV1, L	3.2 (0.8)	5563	−0.14 (−0.24, −0.04)	.01	−0.08 (−0.18, 0.03)	.15
	FVC, L	4.2 (1.0)	5548	−0.16 (−0.27, −0.04)	.01	−0.11 (−0.22, 0.01)	.07

*Note*: In the confounder model, coefficient and 95% confidence interval are adjusted for sex, early life socioeconomic status (parental social class, mother's age at birth, mother's marital status, and mother's education) and early life health (childhood hospitalizations, childhood disability, and internalizing symptoms, and externalizing symptoms). Relationships for systolic blood pressure (SBP) and diastolic blood pressure (DBP) are further separately adjusted for hypertension medication.

Other abbreviations: BMI, body mass index; WHR, waist to hip ratio; CRP, C‐reactive protein (log transformed); HbA1c, glycosylated hemoglobin; HDL, high‐density lipoprotein; IgE, immunoglobulin E (log transformed); LDL, low‐density lipoprotein; tPA, tissue plasminogen activator; vWF, von Willebrand factor; FEV, forced expiratory volume in 1 second; FVC, forced vital capacity; and Cortisol 1, measured 45 minutes after waking (log transformed); Cortisol 2, measured 3h after waking (log transformed). The analytical sample is the non‐missing sample size and is the same for individual models but varies across outcomes.

Next, we examine the influence, if any, of timing, duration, and type of public care exposure on the various biomedical markers depicted in Table 2. There was no support for our hypotheses that entering care later in childhood rather than earlier (Table S2) or having a longer exposure to public care (Table S3) elicited a greater impact on the adult risk markers. Based on the enquiry regarding type of care, of the 144 children looked after by age 11, the majority, 60% (*n* = 86), were or had been in foster care. Type of care was essentially unrelated to our biological indices in later life (Table S4).

## 4 DISCUSSION

The main finding of this prospective birth cohort study was that out‐of‐home care placement in childhood was largely not associated with an array of biomarkers in middle‐age. Given the large number of comparisons necessarily conducted in the course of our analyses, alongside the caveat that not every association explored was hypothesis‐driven, the result for care and lower body mass index is likely to be a chance finding. Thus, although it has been shown that placement in out‐of‐home care during childhood is associated with adverse health outcomes in later adulthood (Gypen et al., 2017) (Zlotnick et al., 2012), including early death (Gao et al., 2017), our findings suggest that the physiological pathway by which our indicator of childhood disadvantage leads to unfavorable health outcomes later in life is unlikely to include the biomarkers examined herein. There is growing evidence that alternative pathways include adult socioeconomic position and mental health (Viner & Taylor, 2005). That childhood psychosocial factors apparently exert a weaker impact on adult biological markers than the outcomes of socioeconomic status, mental health, and health behaviors has been seen in other fields including childhood cognition (Batty et al., 2008; Batty, Deary, Schoon, & Gale, 2007a, 2007b; Calvin et al., 2011).

The strengths of our study included the relatively large study sample, the prospective measurement of public care across childhood as opposed to distant recall in older age which seems to prevail in studies of the long‐term influence of early life adversity, and the comprehensive series of objectively measured biomedical endpoints. Our study of course is not without several limitations. First, 58% of cohort members with out‐of‐home care data took part in the biomedical survey, with participation lower in individuals with such a history (Figure S1). However, provided there was still normal variation in exposure and outcomes in the remaining study members, attrition should have been non‐differential and therefore would not have impacted upon our findings (Batty & Gale, 2009; Batty et al., 2014). Second, parents may provide a socially desirable response to an enquiry as revealing and sensitive as the public care history of their offspring. We are unaware of any studies assessing the agreement of parental self‐reported care with a gold standard such as administrative data. However, in preliminary findings from a meta‐analysis of studies of childhood care exposure and adult mortality risk in which we compared the predictive value of care data as ascertained from parental report with electronic records across studies, we found that care data collected using both modes were similarly associated with around a doubling of adult mortality rates in those with a history of care relative to those without (de Mestral, Bell, & Batty, 2018). Third, vulnerable children and families are hard to reach in a research context, and it is likely that the present study will not capture data on people who experienced the more challenging of upbringings. For that, purposeful sampling of such groups would be required alongside unexposed children to facilitate comparison. Lastly, although we were able to examine the impact of timing, duration, and, to a limited degree, type of placement in out‐of‐home care, we were unable to explore other potentially important placement characteristics, such as reason or stability of out‐of‐home care placement.

In conclusion, our findings suggest that the biomarkers captured in the present study are unlikely to mediate the link between being in care in childhood and later chronic disease or mortality. Other processes, including adult poverty, health behaviors, and mental health, should be further investigated as an alternative pathway.

## CONFLICT OF INTEREST

The authors declare no conflict of interest.

## AUTHOR CONTRIBUTIONS

G.D.B. generated the idea for the paper. G.D.B. and C.d.M. prepared an analytical plan, and C.d.M. conducted the data analyses. G.D.B. with C.d.M. prepared a draft of the manuscript on which all co‐authors commented.

## Supporting information


**Figure S1.** Flow of study members through phases of data collection and in to the present analytical sample, National Child Development Study.
**Table S1.** Beta coefficient (95% confidence interval) for the association of out‐of‐home care in childhood with adult biomedical risk factors, National Child Development Study (*n* = 4029‐5716).
**Table S2.** Multiply‐adjusted beta coefficient (95% confidence interval) for the association of out‐of‐home care placement timing in childhood with adult biomedical risk factors, National Child Development Study (*n* = 4029‐5716).
**Table S3.** Multiply‐adjusted beta coefficient (95% confidence interval) for the association of duration of out‐of‐home care in childhood with adult biomedical risk factors, National Child Development Study (*n* = 3951‐5602).
**Table S4.** Multiply‐adjusted beta coefficient (95% confidence interval) for the association of type of out‐of‐home care in childhood with adult biomedical risk factors, National Child Development Study (*n* = 3685‐5240).
