# Supplementary material for: Out‐of‐home care in childhood and biomedical risk factors in middle‐age: National birth cohort study
Source: Am J Hum Biol. 2019 Nov 5;32(3):e23343. doi: 10.1002/ajhb.23343 (PMC7317568; doi:10.1002/ajhb.23343)
Supplement: Supplementary file 1 — Figure S1. Flow of study members through phases of data collection and in to the present analytical sample, National Child Development Study. Table S1. Beta coefficient (95% confidence interval) for the association of out‐of‐home care in childhood with adult biomedical risk factors, National Child Development Study (n = 4029‐5716). Table S2. Multiply‐adjusted beta coefficient (95% confidence interval) for the association of out‐of‐home care placement timing in childhood with adult biomedical risk factors, National Child Development Study (n = 4029‐5716). Table S3. Multiply‐adjusted beta coefficient (95% confidence interval) for the association of duration of out‐of‐home care in childhood with adult biomedical risk factors, National Child Development Study (n = 3951‐5602). Table S4. Multiply‐adjusted beta coefficient (95% confidence interval) for the association of type of out‐of‐home care in childhood with adult biomedical risk factors, National Child Development Study (n = 3685‐5240). [file AJHB-32-e23343-s001.docx]

**Supplemental Figure 1. Flow of study members through phases of data collection and into the present analytical sample, National Child Development Study**

Intermediate social surveys at age 23 (1981) and 33 (1991)

Complete data on childhood OHC up to age 16 (1965-74)

N=13791

*OHC, out-of-home care*

Analytical sample (complete data on OHC, covariates, mediators & biomedical risk factors)

N=4029-5716

Age 44/45 biomedical survey (2002)

N=8012

Age 44/45 biomedical survey (2002)

N=9377

Age 42 social survey (1999)

N=9663

Age 42 social survey (1999)

N=11419

Perinatal survey (1958)

N=18858

Age 7 social survey (1965)

N=15425

Age 11 social survey (1969)

N=15337

Age 16 social survey (1974)

N=14654

**Supplemental Table 1. Beta coefficient (95% confidence interval) for the association of out-of-home care in childhood**

**with adult biomedical risk factors, National Child Development Study (N=4029-5716)**

|  | **Mean (SD)** | **Analytical sample** | **Model 1** | **p-value** | **Model 2** | **p-value** | **Model 3** | **p-value** | **Model 4** | **p-value** | **Model 5** | **p-value** |
| --- | --- | --- | --- | --- | --- | --- | --- | --- | --- | --- | --- | --- |
| *Cardiovascular disease risk factors* | |  |  |  |  |  |  |  |  |  |  |  |
| Resting SBP, mmHg | 126.4 (16.4) | 5672 | -1.51 (-3.68, 0.65) | 0.17 | -1.43 (-3.63, 0.78) | 0.21 | -1.30 (-3.46, 0.87) | 0.24 | -1.62 (-3.75, 0.50) | 0.14 | -1.17 (-3.35, 1.01) | 0.29 |
| Resting DBP, mmHg | 78.8 (10.8) | 5671 | -0.48 (-1.96, 1.01) | 0.53 | -0.51 (-2.02, 1.00) | 0.51 | -0.33 (-1.81, 1.16) | 0.67 | -0.34 (-1.80, 1.12) | 0.65 | -0.21 (-1.71, 1.29) | 0.78 |
| Resting heart rate, bmp | 71.1 (11.4) | 5702 | 0.50 (-1.12, 2.12) | 0.55 | 0.44 (-1.21, 2.10) | 0.60 | 0.08 (-1.55, 1.70) | 0.93 | -0.22 (-1.82, 1.38) | 0.79 | -0.12 (-1.76, 1.53) | 0.89 |
| BMI, kg/m2 | 27.2 (4.5) | 5616 | -0.44 (-1.08, 0.20) | 0.18 | -0.73 (-1.38, -0.08) | 0.03 | -0.59 (-1.23, 0.05) | 0.07 | -0.44 (-1.08, 0.20) | 0.18 | -0.32 (-0.83, 0.20) | 0.23 |
| WHR | 0.9 (0.1) | 5716 | 0.00 (-0.01, 0.01) | 0.93 | 0.00 (-0.01, 0.01) | 0.57 | 0.00 (-0.01, 0.01) | 0.50 | 0.00 (-0.01, 0.01) | 0.85 | 0.00 (-0.01, 0.01) | 0.51 |
| Triglycerides, mg/L | 1.9 (1.1) | 4755 | -0.01 (-0.18, 0.16) | 0.92 | -0.02 (-0.19, 0.15) | 0.81 | -0.05 (-0.21, 0.13) | 0.61 | -0.04 (-0.20, 0.13) | 0.67 | -0.04 (-0.21, 0.13) | 0.67 |
| HDL, mmol/L | 1.5 (0.4) | 4782 | -0.01 (-0.07, 0.04) | 0.62 | 0.00 (-0.06, 0.06) | 0.99 | 0.00 (-0.06, 0.05) | 0.97 | 0.00 (-0.06, 0.05) | 0.92 | 0.01 (-0.05, 0.06) | 0.80 |
| LDL, mmol/L | 3.4 (0.9) | 4543 | -0.06 (-0.20, 0.08) | 0.39 | -0.06 (-0.20, 0.08) | 0.40 | -0.07 (-0.21, 0.07) | 0.31 | -0.07 (-0.21, 0.07) | 0.31 | -0.07 (-0.21, 0.08) | 0.35 |
| HbA1c, % | 5.2 (0.4) | 4836 | 0.02 (-0.03, 0.08) | 0.42 | 0.01 (-0.05, 0.07) | 0.68 | 0.01 (-0.05, 0.07) | 0.68 | -0.01 (-0.06, 0.05) | 0.87 | -0.01 (-0.06, 0.05) | 0.81 |
| *Inflammatory markers* |  |  |  |  |  |  |  |  |  |  |  |  |
| CRP, mg/L | 1.8 (2.3) | 4720 | 0.02 (-0.16, 0.20) | 0.80 | -0.04 (-0.22, 0.14) | 0.66 | -0.03 (-0.21, 0.15) | 0.77 | -0.02 (-0.19, 0.16) | 0.86 | -0.05 (-0.22, 0.13) | 0.58 |
| Fibrinogen, g/L | 2.9 (0.6) | 4723 | 0.12 (0.03, 0.21) | 0.01 | 0.10 (0.01, 0.19) | 0.03 | 0.09 (0.01, 0.18) | 0.04 | 0.08 (-0.01, 0.16) | 0.08 | 0.07 (-0.02, 0.16) | 0.12 |
| D-dimer, ng/mL | 177.9 (97.9) | 4665 | 4.42 (-10.47, 19.31) | 0.56 | 2.91 (-12.30, 18.11) | 0.71 | 2.77 (-12.24, 17.77) | 0.72 | 2.30 (-12.56, 17.16) | 0.76 | 2.11 (-13.12, 17.34) | 0.79 |
| tPA, ng/mL | 5.1 (2.5) | 4730 | 0.29 (-0.10, 0.68) | 0.14 | 0.19 (-0.21, 0.59) | 0.35 | 0.26 (-0.13, 0.65) | 0.20 | 0.25 (-0.13, 0.63) | 0.20 | 0.22 (-0.17, 0.61) | 0.26 |
| vWF, IU/dL | 120.6 (38.1) | 4732 | 0.47 (-5.53, 6.46) | 0.88 | -0.83 (-6.97, 5.31) | 0.79 | -0.28 (-6.31, 5.76) | 0.93 | -0.48 (-6.48, 5.51) | 0.88 | -1.45 (-7.61, 4.71) | 0.64 |
| IgE, mg/dL | 67.2 (108.9) | 4673 | 0.05 (-0.16, 0.26) | 0.64 | 0.05 (-0.16, 0.27) | 0.63 | -0.01 (-0.22, 0.20) | 0.95 | 0.01 (-0.20, 0.22) | 0.95 | -0.01 (-0.23, 0.21) | 0.93 |
| *Neuroendocrine markers* |  |  |  |  |  |  |  |  |  |  |  |  |
| Cortisol 1, nmol/L | 21.3 (12.2) | 4029 | -0.12 (-0.22, -0.01) | 0.03 | -0.11 (-0.22, -0.01) | 0.03 | -0.10 (-0.21, 0.00) | 0.04 | -0.12 (-0.22, -0.02) | 0.02 | -0.11 (-0.21, -0.01) | 0.04 |
| Cortisol 2, nmol/L | 8.5 (6.9) | 4055 | 0.03 (-0.08, 0.14) | 0.61 | 0.01 (-0.10, 0.12) | 0.82 | 0.02 (-0.09, 0.12) | 0.78 | -0.02 (-0.13, 0.09) | 0.73 | -0.02 (-0.13, 0.09) | 0.68 |
| *Pulmonary function* |  |  |  |  |  |  |  |  |  |  |  |  |
| FEV1, L in 1s | 3.2 (0.8) | 5563 | -0.14 (-0.24, -0.04) | 0.01 | -0.10 (-0.20, 0.01) | 0.06 | -0.11 (-0.21, -0.01) | 0.03 | -0.10 (-0.20, 0.00) | 0.05 | -0.07 (-0.17, 0.04) | 0.20 |
| FVC, L | 4.2 (1.0) | 5548 | -0.16 (-0.27, -0.04) | 0.01 | -0.14 (-0.25, -0.02) | 0.02 | -0.12 (-0.23, -0.01) | 0.04 | -0.13 (-0.24, -0.01) | 0.03 | -0.11 (-0.22, 0.01) | 0.07 |

Comparator is never having experienced out-of-home care. Coefficient and 95% confidence interval are adjusted for: sex (model 1); sex + early life socioeconomic status (parental social class, mother’s age at birth, mother’s marital status, mother’s education) (model 2); sex + early life health (childhood hospitalizations, childhood disability, internalizing symptoms, and externalizing symptoms) (model 3); sex + adult risk factors measured at age 42 year follow-up (occupational social class, body mass index, alcohol intake, and smoking) (model 4); and all above covariates (model 5). Relationships for systolic blood pressure (SBP) and diastolic blood pressure (DBP) are further separately adjusted for hypertension medication. CRP, C reactive protein (log transformed); HbA1c, glycosylated haemoglobin; HDL, high-density lipoprotein; IgE, immunoglobulin E (log transformed); LDL, low-density lipoprotein; tPA, tissue plasminogen activator; vWF, von Willebrand factor; FEV, forced expiratory volume in 1 second; FVC, forced vital capacity; Cortisol 1, measured 45 minutes after waking (log transformed); Cortisol 2, measured 3h after waking (log transformed). The analytical sample is the non-missing sample size and is the same for individual models but varies across outcomes.

**Supplemental Table 2. Multiply-adjusted beta coefficient (95% confidence interval) for the association of out-of-home care placement timing in childhood with adult biomedical risk factors, National Child Development Study (N=4029-5716)**

|  | **Timing of out of home care** | | | | | |
| --- | --- | --- | --- | --- | --- | --- |
|  | **Birth to 7 years** | **p-value** | **Between 7 and 11 years** | **p-value** | **Between 11 and 16 years** | **p-value** |
| *Cardiovascular disease risk factors* | |  |  |  |  |  |
| Resting SBP, mmHg | 1.35 (-1.86, 4.57) | 0.41 | -3.43 (-7.06, 0.20) | 0.06 | -2.67 (-7.12, 1.80) | 0.24 |
| Resting DBP, mmHg | 0.69 (-1.52, 2.90) | 0.54 | -1.70 (-4.19, 0.79) | 0.18 | 0.30 (-2.76, 3.36) | 0.85 |
| Resting heart rate, bmp | -0.62 (-3.04, 1.80) | 0.61 | 0.26 (-2.49, 3.01) | 0.85 | 0.30 (-3.04, 3.64) | 0.86 |
| BMI, kg/m2 | -0.50 (-1.25, 0.25) | 0.19 | -0.41 (-1.27, 0.45) | 0.36 | 0.20 (-0.87, 1.28) | 0.71 |
| WHR | -0.01 (-0.03, 0.00) | 0.04 | 0.00 (-0.02, 0.01) | 0.94 | 0.01 (0.00, 0.03) | 0.12 |
| Triglycerides, mg/L | -0.07 (-0.32, 0.17) | 0.57 | -0.02 (-0.31, 0.28) | 0.92 | 0.00 (-0.35, 0.36) | 0.98 |
| HDL, mmol/L | -0.01 (-0.08, 0.07) | 0.87 | 0.09 (-0.01, 0.18) | 0.07 | -0.08 (-0.19, 0.03) | 0.17 |
| LDL, mmol/L | -0.22 (-0.43, -0.02) | 0.03 | -0.04 (-0.28, 0.20) | 0.76 | 0.21 (-0.08, 0.50) | 0.16 |
| HbA1c, % | 0.06 (-0.02, 0.15) | 0.14 | -0.16 (-0.26, -0.06) | <0.01 | 0.07 (-0.04, 0.19) | 0.20 |
| *Inflammatory markers* |  |  |  |  |  |  |
| CRP, mg/L | -0.09 (-0.35, 0.16) | 0.48 | -0.09 (-0.39, 0.20) | 0.53 | 0.10 (-0.26, 0.46) | 0.58 |
| Fibrinogen, g/L | 0.02 (-0.11, 0.15) | 0.74 | 0.09 (-0.06, 0.24) | 0.23 | 0.13 (-0.04, 0.31) | 0.14 |
| D-dimer, ng/mL | 6.08 (-16.13, 28.30) | 0.59 | 0.52 (-25.29, 26.32) | 0.97 | -3.46 (-34.62, 27.71) | 0.83 |
| tPA, ng/mL | 0.21 (-0.36, 0.78) | 0.47 | 0.15 (-0.51, 0.80) | 0.66 | 0.36 (-0.43, 1.15) | 0.37 |
| vWF, IU/dL | 3.67 (-5.29, 12.63) | 0.42 | -6.55 (-17.13, 4.04) | 0.23 | -4.33 (-16.63, 7.97) | 0.49 |
| IgE, mg/dL | 0.15 (-0.16, 0.46) | 0.34 | -0.30 (-0.67, 0.07) | 0.11 | 0.08 (-0.37, 0.53) | 0.74 |
| *Neuroendocrine markers* |  |  |  |  |  |  |
| Cortisol 1, nmol/L | -0.10 (-0.25, 0.05) | 0.19 | -0.08 (-0.26, 0.10) | 0.38 | -0.17 (-0.39, 0.04) | 0.11 |
| Cortisol 2, nmol/L | -0.05 (-0.21, 0.11) | 0.54 | 0.08 (-0.11, 0.27) | 0.42 | -0.11 (-0.33, 0.12) | 0.34 |
| *Pulmonary function* |  |  |  |  |  |  |
| FEV1, L in 1s | -0.09 (-0.24, 0.07) | 0.26 | -0.04 (-0.22, 0.13) | 0.62 | -0.07 (-0.28, 0.14) | 0.54 |
| FVC, L | -0.13 (-0.30, 0.04) | 0.15 | -0.06 (-0.25, 0.13) | 0.54 | -0.13 (-0.37, 0.10) | 0.27 |

Comparator is never having experienced out-of-home care. Coefficient and 95% confidence interval are adjusted for: sex, early life socioeconomic status (parental social class, mother’s age at birth, mother’s marital status, mother’s education), early life health (childhood hospitalizations, childhood disability, internalizing symptoms, and externalizing symptoms), and adult risk factors measured at age 42 year follow-up (occupational social class, body mass index, alcohol intake, and smoking). Relationships for systolic blood pressure (SBP) and diastolic blood pressure (DBP) are further separately adjusted for hypertension medication. CRP, C reactive protein (log transformed); HbA1c, glycosylated haemoglobin; HDL, high-density lipoprotein; IgE, immunoglobulin E (log transformed); LDL, low-density lipoprotein; tPA, tissue plasminogen activator; vWF, von Willebrand factor; FEV, forced expiratory volume in 1 second; FVC, forced vital capacity; Cortisol 1, measured 45 minutes after waking (log transformed); Cortisol 2, measured 3h after waking (log transformed). The analytical sample is the non-missing sample size and is the same for individual models but varies across outcomes.

**Supplemental Table 3. Multiply-adjusted beta coefficient (95% confidence interval) for the association of duration of out-of-home care in childhood with adult biomedical risk factors, National Child Development Study (N=3951-5602)**

|  | **Duration of out-of-home care** | | | | | |
| --- | --- | --- | --- | --- | --- | --- |
|  | **1 year or less** | **p-value** | **Between 1 and 3 years** | **p-value** | **3 years or more** | **p-value** |
| *Cardiovascular disease risk factors* | |  |  |  |  |  |
| Resting SBP, mmHg | -0.52 (-4.41, 3.37) | 0.79 | 0.31 (-6.31, 6.93) | 0.93 | -4.81 (-10.47, 0.86) | 0.10 |
| Resting DBP, mmHg | -0.65 (-3.32, 2.02) | 0.63 | -0.92 (-5.46, 3.63) | 0.69 | -4.66 (-8.55, -0.77) | 0.02 |
| Resting heart rate, bmp | -0.62 (-3.56, 2.33) | 0.68 | 1.53 (-3.49, 6.55) | 0.55 | -3.24 (-7.54, 1.05) | 0.14 |
| BMI, kg/m2 | 0.03 (-0.91, 0.96) | 0.96 | -0.36 (-1.96, 1.24) | 0.66 | -1.06 (-2.39, 0.27) | 0.12 |
| WHR | 0.00 (-0.01, 0.02) | 0.91 | 0.01 (-0.02, 0.04) | 0.45 | -0.02 (-0.04, 0.01) | 0.13 |
| Triglycerides, mg/L | -0.05 (-0.37, 0.27) | 0.76 | -0.06 (-0.60, 0.48) | 0.82 | -0.35 (-0.81, 0.11) | 0.13 |
| HDL, mmol/L | -0.09 (-0.18, 0.01) | 0.09 | -0.02 (-0.19, 0.15) | 0.82 | 0.06 (-0.08, 0.20) | 0.40 |
| LDL, mmol/L | 0.02 (-0.25, 0.28) | 0.90 | 0.22 (-0.24, 0.68) | 0.34 | -0.13 (-0.52, 0.25) | 0.50 |
| HbA1c, % | 0.11 (0.00, 0.21) | 0.04 | 0.08 (-0.09, 0.26) | 0.35 | -0.03 (-0.18, 0.12) | 0.67 |
| *Inflammatory markers* |  |  |  |  |  |  |
| CRP, mg/L | -0.13 (-0.45, 0.20) | 0.44 | 0.52 (0.01, 1.03) | 0.04 | -0.32 (-0.79, 0.14) | 0.18 |
| Fibrinogen, g/L | 0.15 (-0.01, 0.31) | 0.07 | 0.22 (-0.03, 0.47) | 0.09 | -0.08 (-0.31, 0.14) | 0.47 |
| D-dimer, ng/mL | 16.69 (-11.16, 44.55) | 0.24 | -10.29 (-55.81, 35.22) | 0.66 | -26.55 (-67.23, 14.12) | 0.20 |
| tPA, ng/mL | 0.24 (-0.48, 0.95) | 0.51 | 0.35 (-0.79, 1.48) | 0.55 | 0.63 (-0.38, 1.65) | 0.22 |
| vWF, IU/dL | -2.86 (-14.13, 8.42) | 0.62 | 12.70 (-5.19, 30.59) | 0.16 | 8.62 (-7.47, 24.71) | 0.29 |
| IgE, mg/dL | 0.00 (-0.39, 0.38) | 0.99 | -0.33 (-1.02, 0.36) | 0.34 | 0.12 (-0.47, 0.72) | 0.69 |
| *Neuroendocrine markers* |  |  |  |  |  |  |
| Cortisol 1, nmol/L | -0.05 (-0.24, 0.13) | 0.57 | -0.23 (-0.55, 0.08) | 0.15 | -0.06 (-0.33, 0.22) | 0.68 |
| Cortisol 2, nmol/L | -0.05 (-0.25, 0.15) | 0.63 | 0.11 (-0.21, 0.44) | 0.50 | -0.07 (-0.37, 0.22) | 0.63 |
| *Pulmonary function* |  |  |  |  |  |  |
| FEV1, L in 1s | 0.01 (-0.17, 0.20) | 0.90 | -0.17 (-0.49, 0.15) | 0.29 | -0.16 (-0.43, 0.12) | 0.27 |
| FVC, L | -0.05 (-0.25, 0.16) | 0.66 | -0.14 (-0.50, 0.22) | 0.45 | 0.00 (-0.31, 0.31) | 0.99 |

Comparator is never having experienced out-of-home care. Coefficient and 95% confidence interval are adjusted for: sex, early life socioeconomic status (parental social class, mother’s age at birth, mother’s marital status, mother’s education), early life health (childhood hospitalizations, childhood disability, internalizing symptoms, and externalizing symptoms), and adult risk factors measured at age 42 year follow-up (occupational social class, body mass index, alcohol intake, and smoking). Relationships for systolic blood pressure (SBP) and diastolic blood pressure (DBP) are further separately adjusted for hypertension medication. CRP, C reactive protein (log transformed); HbA1c, glycosylated haemoglobin; HDL, high-density lipoprotein; IgE, immunoglobulin E (log transformed); LDL, low-density lipoprotein; tPA, tissue plasminogen activator; vWF, von Willebrand factor; FEV, forced expiratory volume in 1 second; FVC, forced vital capacity; Cortisol 1, measured 45 minutes after waking (log transformed); Cortisol 2, measured 3h after waking (log transformed). The analytical sample is the non-missing sample size and is the same for individual models but varies across outcomes.

**Supplemental Table 4. Multiply-adjusted beta coefficient (95% confidence interval) for the association of type of out-of-home care in childhood with adult biomedical risk factors, National Child Development Study (N=3685-5240)**

|  | **Type of out-of-home care** | | | |
| --- | --- | --- | --- | --- |
|  | **Other care** | **p-value** | **Foster care** | **p-value** |
| *Cardiovascular disease risk factors* |  |  |  |  |
| Resting SBP, mmHg | 2.73 (-2.23, 7.70) | 0.28 | 0.67 (-3.54, 4.89) | 0.75 |
| Resting DBP, mmHg | 1.74 (-1.65, 5.14) | 0.32 | 0.06 (-2.83, 2.94) | 0.97 |
| Resting heart rate, bmp | 2.08 (-1.70, 5.85) | 0.28 | -2.57 (-5.74, 0.61) | 0.11 |
| BMI, kg/m2 | 0.43 (-0.74, 1.60) | 0.47 | -1.19 (-2.17, -0.21) | 0.02 |
| WHR | 0.00 (-0.02, 0.02) | 0.95 | -0.02 (-0.04, -0.01) | 0.01 |
| Triglycerides, mg/L | 0.15 (-0.23, 0.54) | 0.44 | -0.22 (-0.53, 0.10) | 0.18 |
| HDL, mmol/L | -0.08 (-0.20, 0.04) | 0.17 | 0.05 (-0.05, 0.15) | 0.33 |
| LDL, mmol/L | -0.08 (-0.40, 0.24) | 0.63 | -0.32 (-0.58, -0.06) | 0.02 |
| HbA1c, % | 0.11 (-0.02, 0.24) | 0.09 | 0.02 (-0.09, 0.13) | 0.73 |
| *Inflammatory markers* |  |  |  |  |
| CRP, mg/L | 0.03 (-0.37, 0.43) | 0.88 | -0.20 (-0.53, 0.13) | 0.23 |
| Fibrinogen, g/L | 0.00 (-0.20, 0.20) | 0.99 | 0.04 (-0.13, 0.20) | 0.68 |
| D-dimer, ng/mL | 29.05 (-5.49, 63.59) | 0.10 | -11.53 (-40.36, 17.29) | 0.43 |
| tPA, ng/mL | 0.97 (0.10, 1.85) | 0.03 | -0.27 (-1.00, 0.46) | 0.47 |
| vWF, IU/dL | -1.64 (-15.54, 12.26) | 0.82 | 6.38 (-5.10, 17.86) | 0.28 |
| IgE, mg/dL | 0.33 (-0.17, 0.82) | 0.20 | 0.03 (-0.36, 0.43) | 0.86 |
| *Neuroendocrine markers* |  |  |  |  |
| Cortisol 1, nmol/L | -0.14 (-0.37, 0.10) | 0.25 | -0.03 (-0.23, 0.16) | 0.74 |
| Cortisol 2, nmol/L | -0.06 (-0.30, 0.19) | 0.66 | -0.06 (-0.27, 0.15) | 0.60 |
| *Pulmonary function* |  |  |  |  |
| FEV1, L in 1s | -0.05 (-0.29, 0.19) | 0.69 | -0.11 (-0.31, 0.08) | 0.26 |
| FVC, L | -0.21 (-0.48, 0.06) | 0.12 | -0.06 (-0.28, 0.17) | 0.63 |

Comparator is never having experienced out-of-home care. Coefficient and 95% confidence interval are adjusted for: sex, early life socioeconomic status (parental social class, mother’s age at birth, mother’s marital status, mother’s education), early life health (childhood hospitalizations, childhood disability, internalizing symptoms, and externalizing symptoms), and adult risk factors measured at age 42 year follow-up (occupational social class, body mass index, alcohol intake, and smoking). Relationships for systolic blood pressure (SBP) and diastolic blood pressure (DBP) are further separately adjusted for hypertension medication. CRP, C reactive protein (log transformed); HbA1c, glycosylated haemoglobin; HDL, high-density lipoprotein; IgE, immunoglobulin E (log transformed); LDL, low-density lipoprotein; tPA, tissue plasminogen activator; vWF, von Willebrand factor; FEV, forced expiratory volume in 1 second; FVC, forced vital capacity; Cortisol 1, measured 45 minutes after waking (log transformed); Cortisol 2, measured 3h after waking (log transformed). The analytical sample is the non-missing sample size and is the same for individual models but varies across outcomes.
